# *Asplenium paucipinnatum* (Aspleniaceae), a new fern species from southern Thailand, based on morphological and molecular data

**DOI:** 10.3897/phytokeys.272.173645

**Published:** 2026-04-02

**Authors:** Ke-Wang Xu, Rossarin Pollawatn, Liang Zhang, Xin-Mao Zhou, Li-Bing Zhang

**Affiliations:** 1 Co-Innovation Center for Sustainable Forestry in Southern China, Key Laboratory of National Forestry and Grassland Administration on Subtropical Forest Biodiversity Conservation, College of Life Science, Nanjing Forestry University, Nanjing 210037, China Yunnan University Kunming China; 2 Plant of Thailand Research Unit, Department of Botany, Faculty of Science, Chulalongkorn University, Bangkok 10330, Thailand Chulalongkorn University Bangkok Thailand; 3 Key Laboratory for Plant Diversity and Biogeography of East Asia, Kunming Institute of Botany, Chinese Academy of Sciences, Kunming 650201, China Kunming Institute of Botany, Chinese Academy of Sciences Kunming China; 4 Laboratory of Ecology and Evolutionary Biology, State Key Laboratory for Conservation and Utilization of Bio Resources in Yunnan, Yunnan University, Kunming 650091, China Nanjing Forestry University Nanjing China; 5 Missouri Botanical Garden, 4344 Shaw Blvd, St. Louis 63110, MO, USA Missouri Botanical Garden St. Louis United States of America

**Keywords:** *Asplenium aethiopicum* clade, fern phylogeny, Southeast Asian fern flora, taxonomy

## Abstract

*Asplenium paucipinnatum*, a new fern species from Nakhon Si Thammarat, Thailand, is described. Morphologically, it closely resembles *A. micantifrons* in having short-creeping and radial rhizome steles, 1-pinnate laminae, deeply incised pinnae on the basiscopic margin, and sori arranged in two rows close to the costae. However, *A. paucipinnatum* is readily distinguished by having fewer lateral pinnae (< 14 pairs), obliquely rhombic pinnae with acute apices and obtuse marginal teeth, contrasting the more numerous pinnae (> 15 pairs), lanceolate pinnae with acuminate apices, and acute teeth in *A. micantifrons*. Phylogenetic analyses based on multiple plastid DNA regions (*atpB*, *rbcL*, *rps4*, *rps4-trnS*, *trnL*, and *trnL-F*) support *A. paucipinnatum* as a distinct lineage, forming a sister relationship with a clade containing *A. lepturus* and *A. contiguum*. The combination of morphological and molecular evidence supports the recognition of this new taxon. Ecological notes, geographic distribution, and a comparison with related species are also provided.

## Introduction

The fern genus *Asplenium* L. (Aspleniaceae), a prominent member of the Aspleniineae suborder of leptosporangiate ferns, comprises more than 700 extant species worldwide (Hassler 1994–2025; Lin and Viane 2013; PPG I 2016). The genus is characterized by erect or short-creeping rhizomes with a radial stelar structure, rachises that are distinctly sulcate with a central raised ridge flanked by two lateral grooves on each side, and a distinctive rachis-costa architecture featuring a terete, alate rachis whose wings are confluent with the basiscopic margin of the pinnae (Murakami and Moran 1993; Lin and Viane 2013; Xu et al. 2020). In East and Southeast Asia, the vast majority of *Asplenium* species possess erect rhizomes, with only a few exceptions such as *A. batuense* Alderw., *A. micantifrons* (Tuyama) Tuyama ex H.Ohba, and *A. lepturus* J.Sm. ex C.Presl having short-creeping rhizomes (Hassler 1994–2025; Lindsay and Middleton 2012; Lin and Viane 2013). In Thailand, a total of 43 species of *Asplenium* have been recorded, including two recently described species, *A. appressifolium* Boonkerd & Petchsri and *A. minutifolium* Kanem. & Tagane (Boonkerd and Pollawatn 2000; Boonkerd 2009; Lindsay and Middleton 2012; Lindsay et al. 2013; Kanemitsu et al. 2017; Petchsri and Boonkerd 2021).

In July 2019, during a botanical survey in Khao Luang National Park, Nakhon Si Thammarat Province, southern Thailand, an unusual *Asplenium* gathering was collected. Morphologically, it showed similarities to species such as *A. micantifrons* from Japan in rhizome habit and pinna dissection but differed in having fewer lateral pinnae (< 14 pairs), obliquely rhombic pinnae with acute apices and obtuse marginal teeth. Comprehensive morphological and phylogenetic analyses confirmed its status as a distinct new species, which is described herein as *A. paucipinnatum*.

## Materials and methods

### Materials and taxon sampling

**Figure 1.**
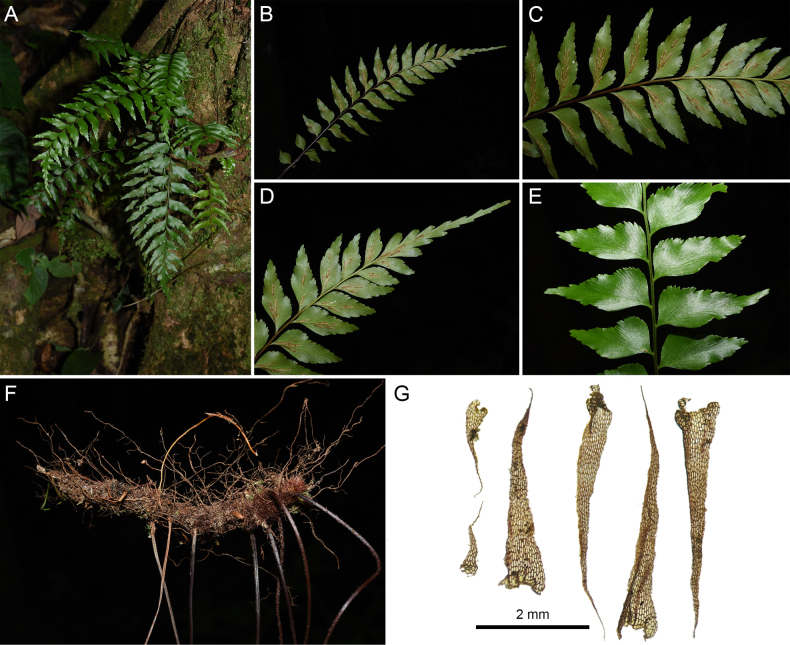
*Asplenium paucipinnatum*. **A**. Habit; **B**. Abaxial view of lamina; **C**. Abaxial view of pinnae showing the venation on the pinnae; **D**. Showing the lamina apices deeply pinnatifid and becoming gradually decrescent upwards; **E**. Adaxial view of Pinnae at middle portion of the laminae; **F**. Short-creeping rhizome; **G**. Rhizome scales. (Voucher specimen: *Li Bing Zhang, Liang Zhang, R. Pollawatn & X. M. Zhou 10976*).

This study employed examinations of the holotype and isotype specimens, together with field observations, for the morphological characterization of the new species (Fig. 1). The new species was hypothesized to be a member of the *A. aethiopicum* subclade (Xu et al. 2020), based on a suite of morphological characters comprising short-creeping rhizomes (densely scaled at the apex), 1-pinnate laminae, and thinly coriaceous pinnae. To verify this placement phylogenetically, we followed the phylogenetic framework of Xu et al. (2020) and incorporated sequence data from the new taxon into their dataset for the *A. aethiopicum* subclade. The ingroup comprised 43 accessions representing 31 *Asplenium* species of the *A. aethiopicum* subclade. Two species of *Asplenium* of the *A. ensiforme* subclade were used as outgroups based on a previous study of the family (Xu et al. 2020).

#### DNA extraction, PCR amplification and sequencing

Total genomic DNA was extracted from silica-gel-dried leaf material using a modified CTAB method (Doyle and Doyle 1987). We amplified and sequenced six plastid DNA regions: the *atpB* gene, the *rbcL* gene, the *rps4* gene, the *rps4-trnS* intergenic spacer, the *trnL* intron, and the *trnL-F* intergenic spacer. The primer sequences and PCR protocols employed in this study were conducted as described in previously established references. Amplification of the *rbcL* gene was performed using primers ESRBCL1F (ATGTCACCACAAACGGAGACTAAAGC) and ESRBCL1361R (TCAGGACTCCACTTACTAGCTTCACG), while the *atpB* gene was amplified with primers ESATPB172F (AATGTTACTTGTGAAGTWCAACAAT) and ESATPE45R (ATTCCAAACWATTCGATTWGGAG), both sets designed by Schuettpelz and Pryer (2007). The *rps4* gene along with the *rps4–trnS* intergenic spacer were amplified with the forward primer ATGTCMCGTTAYCGAGGRCCTCGT and the reverse primer TACCGAGGGTTCGAATC, following Schneider et al. (2005). For the *trnL* intron and the *trnL–F* intergenic spacer, amplification was carried out using primer F (ATTTGAACTGGTGACACGAG) from Taberlet et al. (1991) and primer Fern1 (GGCAGCCCCCARATTCAGGGRAACC) from Trewick et al. (2002). PCR cycling conditions were as described in Xu et al. (2020). The PCR products were purified using TIANquick mini purification kits (TIANGEN, Beijing, China) and sequenced by Sangon Biotech Co., Ltd. (Shanghai, China). Four sequences from one accession of the new species were newly generated; others were obtained from GenBank. All sequences, along with their corresponding GenBank accession numbers and voucher information, are provided in Suppl. material 1.

#### Sequence alignment and phylogenetic analysis

Sequence editing and assembly were performed using Sequencher v.4.14 (GeneCodes Corporation, Ann Arbor, Michigan). The resulting sequences were aligned and manually adjusted with BioEdit (Hall 1999). Phylogenetic analyses were conducted using Bayesian inference (BI) and maximum likelihood (ML) criteria. BI was performed in MrBayes 3.2.7 (Huelsenbeck and Ronquist 2001). Two independent Markov chain Monte Carlo (MCMC) analyses were performed in parallel, each consisting of four chains run for 10 million generations. This run length was sufficient for the standard deviation of split frequencies to fall below 0.01, indicating convergence of the chains. Trees were sampled every 1,000 generations. jModelTest 2.1.6 (Darriba et al. 2012) was used to select the best-fitting mode for BI and ML analyses. Substitution models were assigned separately to each partition based on the specific evolutionary patterns of each marker as follows: a TIM1+G model was applied to *rbcL*, a TIM3+G model to *atpB*, a TVM+G model to the *rps4* gene and *rps4–trnS* spacer, and a TPM1uf+G model to the *trnL* intron and the *trnL–F* intergenic spacer. Default priors were used, and all substitution model parameters were unlinked across loci. Tracer v1.5 (Rambaut et al. 2018) was employed to assess stationarity and determine an appropriate burn-in period. After discarding burn-in samples, the remaining trees from both runs were combined to construct a 50% majority-rule consensus topology, with nodal support evaluated using posterior probabilities (BIPP). ML analysis was conducted using RAxML v.8, which performed 1000 rapid bootstrap replicates followed by a search for the best-scoring tree in a single run. Both ML and BI analyses were conducted on Cipres (Miller et al. 2010). The best-fit model GTR+G for the combined dataset for BI and ML was selected under the Akaike Information Criterion (AIC) using jModelTest2 v.2.1.6 (Darriba et al. 2012).

## Results and discussion

**Figure 2.**
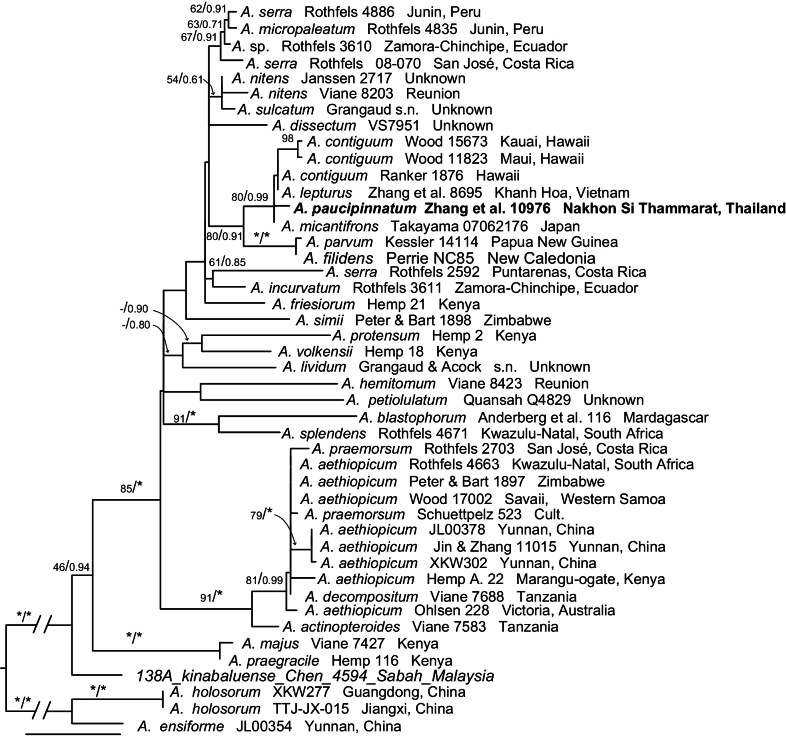
The phylogenetic position of *Asplenium paucipinnatum* based on four plastid markers (*atpB*, *rbcL*, *rps4*, *rps4-trnS*, *trnL*, and *trnL-F*). The numbers associated with branches are maximum likelihood bootstrap support (MLBS) and Bayesian posterior probability (BIPP). The asterisk indicates MLBS = 100, BIPP = 1.00.

The combined plastid DNA matrix was 4,297 bp in length, containing 375 variable characters, of which 184 were parsimony-informative. Given that the BI and ML trees were largely congruent aside from some poorly supported branches, the ML tree was used as the basis for the phylogenetic reconstruction presented in Fig. 2. Based on the phylogenetic analysis of six plastid DNA regions (*atpB*, *rbcL*, *rps4*, *rps4-trnS*, *trnL*, and *trnL-F*), the broader phylogenetic relationships within the *Asplenium aethiopicum* subclade remain only partially resolved and often receive low to moderate branch support (Fig. 2). The limited resolution and variable support values across the tree, similar to the results reported by Xu et al. (2020), highlight the complex evolutionary history of this group. It is important to note that the phylogenetic hypothesis presented here, based on only six plastid markers, may not fully capture the species relationships across the tree. This limitation could stem from a combination of insufficient phylogenetic signal and incomplete sequence data for some taxa obtained from public databases (e.g., *A. micantifrons* was represented by only the *rbcL* sequence). Despite this, *A. paucipinnatum* is resolved as a distinct lineage, forming a well-supported clade (MLBS = 80%, BIPP = 0.99) with *A. contiguum*, *A. micantifrons*, and *A. lepturus*. This relationship suggests a close evolutionary affinity among the four species, which is consistent with their shared morphological characteristics such as short-creeping rhizomes, 1-pinnate laminae, and sori borne in two rows closely set along to the costa. Interestingly, we observed that *A. paucipinnatum* differs from its close relative *A. micantifrons* by only a single base pair in the *rbcL* gene. Despite this minimal molecular divergence in this particular marker, the two species exhibit substantial morphological differences: *A. paucipinnatum* differs in its fewer pinna pairs, as well as its broader, obliquely rhombic pinnae that lack a caudate apex.

Within *Asplenium*, the short-creeping rhizome habit is uncommon and is largely restricted to the *A. aethiopicum* subclade (Xu et al. 2020). It is noteworthy that the species within this subclade which share this rhizome type usually exhibit a consistent combination of traits: densely scaly rhizomes, narrow and caudate pinnae, and a peculiar perispore pattern (Lin and Viane 2013). However, *A. paucipinnatum* exhibits a distinct morphology compared to its relatives in the subclade, most notably in its fewer pinna pairs, as well as its broader, obliquely rhombic pinnae that lack a caudate apex (Fig. 1). In terms of distribution, this group is predominantly found in Africa and Central/South America. In contrast, its presence in Asia is limited to a small number of species: the Japanese endemic *A. micantifrons*, the Southeast Asian *A. lepturus*, and the Pacific-island species *A. contiguum* (also found in northeastern Thailand), the New Caledonia *A. filidens* Brownlie, and *A. parvum* Watts, which ranges from Papua New Guinea to northeastern Queensland and New Caledonia (Lindsay and Middleton 2013). Our study reveals a strong concordance between biogeographic distribution and phylogenetic relationships. Specifically, the Asian species form a well-supported, distinct monophyletic lineage in our phylogenetic tree (Fig. 2).

### Taxonomic treatment

#### Asplenium paucipinnatum

Taxon classificationPlantaePolypodialesAspleniaceae

K.W.Xu, Li Bing Zhang & Pollawatn sp. nov.

673C7CB7-8FBA-5C24-9477-9E2FA1D4379D

urn:lsid:ipni.org:names:77378261-1

Fig. 1

##### Type.

Thailand • Nakhon Si Thammarat: Phrom Khiri District, Khao Luang National Park, elev. 300–600 m, 8°31'17"N, 99°46'43"E, 23 July 2019, *Li Bing Zhang. Liang Zhang, R. Pollawatn & X.M. Zhou 10976* (holotype: BCU-P05253; isotypes: CDBI, KUN, MO, PYU).

##### Description.

*Asplenium paucipinnatum* resembles *A. micantifrons* (Tuyama) Tuyama ex H.Ohba by its short creeping and radial rhizomes, 1-pinnate laminae, pinnae deeply incised at basiscopic side of margin, and sori borne in 2 rows closely set along to the costa, but the former has lateral pinnae fewer than 14 pairs, pinnae oblique rhombic, pinna apex acute, and marginal teeth obtuse. In contrast, *A. micantifrons* has lateral pinnae more than 15 pairs, pinnae lanceolate, pinna apex acuminate, and marginal teeth acute.

***Plants*** low-epiphytic or less often terrestrial, often on mossy tree trunks, occasionally in moist rock crevices, 20–40 cm tall. ***Rhizomes*** short creeping, with radial steles, up to 5 mm diameter, with fronds 2–6 mm apart, densely covered with scales. ***Rhizomes scales*** reddish brown, thickly membranaceous, narrowly triangular, margins slightly denticulate, (1.2–)3.5–4.5(–5.5) × (0.2–)0.55–0.65(–0.75) mm. ***Fronds*** widely spaced, not proliferous, 20–40 cm long, thinly coriaceous. ***Stipes*** semi-shiny, purplish to dark brown, up to 13 cm long, with few brown lanceolate small scales similar to those on the rhizome. ***Laminae*** oblong to elliptic in outline, 18–27 × 6–10 cm, 1-pinnate, acuminate, basal pinnae somewhat reduced, apical segment deeply pinnatifid or gradually decrescent. ***Rachises*** dark brown at base of laminae, gradually changing to stramineous at top of laminae, subglabrous, terete abaxially, adaxial side grooved and with 2 green narrow wings. ***Pinnae*** 10–14 pairs, basal pinnae subopposite, others alternate, middle ones larger, obliquely spreading, (shiny) dark green adaxially, pale green abaxially, obliquely rhomboid, deeply incised at basiscopic side of margin, (2–)2.5–4 × 0.6–1 cm, mostly shortly petiolate, unequally cuneate at the base tending to be slightly auriculate acroscopically, apex acute, coarsely serrate or occasionally bearing a lobe at acroscopical pinnae with lobes up to 2/3 of the way to the costa. ***Veins*** free, visible on both surfaces, simple, once or twice forked, not reaching pinnae margins. ***Sori*** (2–)5–9 per pinna, linear, ca. 5 mm long, borne in two closely set rows along the costa. ***Indusia*** linear, membranous, entire, ± 1 mm wide, persistent.

##### Distribution and habitat.

*Asplenium paucipinnatum* is currently known only from one locality in Khao Luang National Park, Phrom Khiri District, Nakhon Si Thammarat, Thailand. It was observed to grow on moss-covered tree trunks or crevices of moist rocks within tropical rainforests at an elevation of 300–600 m.

##### Conservation and IUCN Red List category.

This species is currently known only from its type locality, where a single population comprising fewer than 50 individuals has been observed. According to the IUCN Red List guidelines, it is therefore provisionally assessed as Critically Endangered (CR) under criterion D. Further field surveys are required to determine whether this species occurs in other regions.

##### Etymology.

The specific epithet “*paucipinnatum*” alludes to the paucity of pinnae in comparison to those of its closely related species with short-creeping rhizome.

## Supplementary Material

XML Treatment for Asplenium paucipinnatum

## Additional information

### Use of AI

No use of AI was reported.

### Author ORCIDs

Ke-Wang Xu https://orcid.org/0000-0003-2239-0487

Xin-Mao Zhou https://orcid.org/0000-0003-3555-7784

Li-Bing Zhang https://orcid.org/0000-0002-4905-040X

### Data availability

All of the data that support the findings of this study are available in the main text or Supplementary Information.

## Supplementary materials

Supplementary material 1 List of voucher specimens and Genbank accession numbers used in phylogenetic analyses - indicates missing data Data type xlsx https://binary.pensoft.net/file/1578905This dataset is made available under the Open Database License (http://opendatacommons.org/licenses/odbl/1.0/). The Open Database License (ODbL) is a license agreement intended to allow users to freely share, modify, and use this Dataset while maintaining this same freedom for others, provided that the original source and author(s) are credited. Ke-Wang Xu, Rossarin Pollawatn, Liang Zhang, Xin-Mao Zhou, Li-Bing Zhang
